# Non-Technical Loss Detection in Power Grids with Statistical Profile Images Based on Semi-Supervised Learning

**DOI:** 10.3390/s20010236

**Published:** 2019-12-31

**Authors:** Jiangteng Li, Fei Wang

**Affiliations:** School of Electronic Information and Communications, Huazhong University of Science and Technology, Wuhan 430074, China; lijiangteng@hust.edu.cn

**Keywords:** sensor system, smart meter, non-technical loss, deep learning, semi-supervised learning

## Abstract

In order to keep track of the operational state of power grids, the world’s largest sensor system, smart grid, was built by deploying hundreds of millions of smart meters. Such a system makes it possible to discover and make quick response to any hidden threat to the entire power grid. Non-technical losses (NTLs) have always been a major concern for their consequent security risks as well as immeasurable revenue loss. However, various causes of NTL may have different characteristics reflected in the data. Accurately capturing these anomalies faced with such a large scale of collected data records is rather tricky as a result. In this paper, we proposed a new methodology of detecting abnormal electricity consumptions. We did a transformation of the collected time-series data which turns it into an image representation that could well reflect users’ relatively long term consumption behaviors. Inspired by the excellent neural network architecture used for objective detection in computer vision, we designed our deep learning model that takes the transformed images as input and yields joint features inferred from the multiple aspects the input provides. Considering the limited amount of labeled samples, especially the abnormal ones, we used our model in a semi-supervised fashion that was brought about in recent years. The model is tested on samples which are verified by on-field inspections and our method showed significant improvement for NTL detection compared with the state-of-the-art methods.

## 1. Introduction

By deploying a large number of sensor devices, the smart grid becomes the largest sensor network in the world. Particularly, China installed nearly 500 million smart meters (SM) by the end of 2017. The smart meters have greatly improved the information and automation level of the power grid, and made it possible to identify anomalies. Anomalies, which include meter failure and electricity theft, are the primary source of non-technical losses (NTL) in the power grid. The NTL cause significant revenue losses; $58.7 billion dollars are lost each year due to NTL [1]. Further, it also affects the power system operation because of the uncertainty of the real consumption [2]. Due to the huge scale of the collected data and the complicated real-world operational environment, the NTL detecting methods required are excellent in terms of both efficiency and reliability. However, the existing expert system based approach sometimes is unreliable as it fails to comprehensively exploit the underlying correlations among different features. Or the other way round, in order to cover extremely diverse forms of abnormal behaviors, far more field knowledge should be integrated, and sometimes human efforts are necessary. As a result, efficiency suffers.

In this paper, we introduced deep learning to help with the detection of NTL as it has already demonstrated its great potential and capacity in a variety of fields. Neural networks’ hierarchical structures are designed to automatically extract high level features containing more abstract semantic information from a large scale of input data. So, in this problem setting where the prominent features are latent and intertwined with a lot of factors, deep neural networks are just fit for the job in terms of both efficiency and accuracy. Yet, there are some problems we have to solve:

(a) The quality of collected data is relatively low because of data noise and missing data. As is often the case in real-world scenarios, the data records may suffer from various types of noises. Generally, two types of noises are distinguished: feature (or attribute) noise and class noise. Class noise means the labels assigned to samples are not totally correct, which is likely to happen as the ground truth labels are given based on the result of on-field inspections in this problem. In this paper we only focus on the feature noises. Generally speaking, this type of noise can be summarized as the noise that interferes the mapping from the input *x* to the output label *y*, including slight value disturbance, erroneous data records as well as data out of sync. Missing data is also a common phenomenon in large-scale sensing systems. Situations get worse when unavailable data points emerge in high density or even large chunks are missing.
(b) The correlation between labels given by on-field inspection and the original data records is not intuitional. For those verified customers, we only have general labels denoting whether anomalies ever occurred in a customers’ history records. There is no further information that indicates when and how faults or fraudulent events occurred. So we have to find distinct patterns of positive/negative samples for the classifier/detector. It is better if we can make the data records more human friendly and distinguishable for ordinary people without the aid of carefully designed criteria.
(c) The proportion of labeled data is rather small compared to that of the unlabeled. And the abnormal samples are obviously fewer than normal ones. This fact makes it impossible for us to treat the problem as a simple supervised classification problem.

In this paper, we first proposed a data transformation method. By analyzing the abnormal cases, we found that statistical characteristics of the data can better reflect the consumption behaviors of the electricity users. The method we designed focuses on the feature patterns in a relatively longer term by utilizing the data in a certain time range. This transformation makes it easier to get features that can distinguish between normal and abnormal samples. Moreover, the transformed data form naturally has better noise resistance performance and it can eliminate the difficulties resulting from different value ranges of different users. We also adopt a semi-supervised model [3] brought about in recent years. This model does not depend on any presupposed prior distribution of the training data in class space and proved itself a reliable one by testing it on the real customers’ data.

There is an abundance of previous work about NTL detection or anomaly (fraud) detection in electricity industry. The approaches can generally be categorized in classification based and non-classification based solutions. The non-classification based approaches usually use methods like clustering, statistical analysis, and so on. Ford et al. [4] and Cody et al. [5] forecasted the customers’ energy consumption. If the difference between actual and forecasted consumption exceeds the limit imposed by the authors, the customer is considered to be committing fraud. Similar ideas are adopted in [6,7], where researchers first construct the distribution of the historical data collected by meters. Then a similarity measurement is introduced to find outliers, which are considered fraud or anomaly. As far as the classification based approaches are concerned, Nagi et al. [8] used extracted features from historical customer consumption data and trained a support vector machine (SVM) model with them to detect NTL. Apart from the sole consumption measurements, P. Glauner et al. [9] also utilized the customers’ geographical location to compute the inspection rate and the NTL rate in its neighborhood. And other models besides support vector machine (SVM), such as logistic regression (LR), k-nearest neighbor (kNN), and random forest (RF) are trained to testify the methodology. As for the classifier, there also exists works using neural networks (NN) as in [10,11]. However, reference [11] did a comparison among different algorithms and found out the single gradient boosted machine (GBM) outperformed any ensemble or any other classifiers.

Our main contributions to these problems are as follows:

- We designed a novel way of visualizing customers’ data records by utilizing more potentially useful real-time electrical information than sole energy consumption measurements. The new data format statistically constructs a visual representation of customers’ behavior in a certain time range and provides a new aspect of viewing their operational states. Such transformation is also tolerant of slight data error or missing, which is quite common for the sensor systems in industrial areas. The convolutional based neural network that is universally used in computer vision is then introduced to extract visual patterns for further classification.
- The inefficient on-field inspection limited the ground truth we can make use of for supervised training. This work combined a semi-supervised learning method based on consistency loss with our proposed network architecture to deal with the common situations in real world scenarios where the training samples are partially labeled. It turns out the consequent overfitting problem can be addressed pretty well.

## 2. Data Transformation and Network Design 

The major goal of this paper is to detect abnormal energy consumption for a certain customer from SM data, whatever it is caused by metering failure or fraudulent usage. The data records are from an electrical information collection system constructed by China Southern Power Grid Co., Ltd. (CSG).

**Figure 1:**
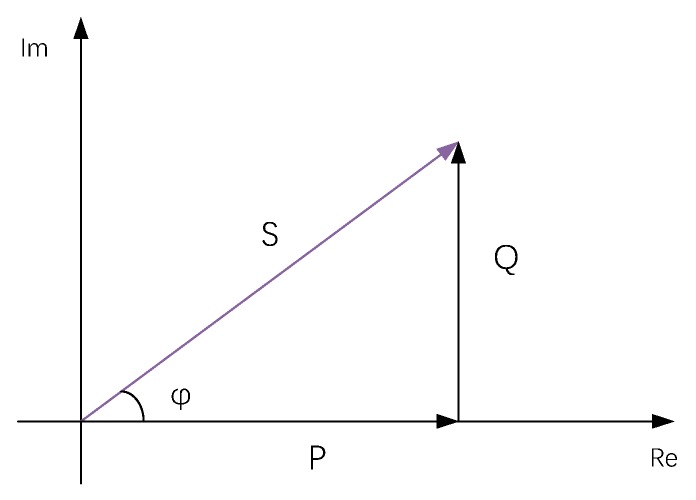
The power triangle in the AC circuit. S is the complex power and the magnitude of S is called apparent power. Active power P is represented as the real axis while reactive power is represented as the imaginary axis of the vector diagram. φ is the angle of difference (in degrees) between current and voltage.

We first extracted features from the electric parameters. Then we did a data transformation and formed training samples based on those features. An inference network was designed afterwards and was trained in a semi-supervised fashion considering the fact that the Figure 1 labeled samples are in the minority.

### 2.1. Statistical Profile

In an alternating current (AC) circuit, electric parameters like voltage, current, etc., are usually denoted in phasor form. The definition of power is more complex than that in the direct current circuit.

(1) $$S=VI^{*}=\left|S\right|∠\varphi$$

*S* is the complex power and its magnitude is the apparent power. *V* denotes voltage in phasor form and *I* denotes current in phasor form. And the complex conjugate of *I* is denoted $I^{*}$. *φ* is the angle of difference (in degrees) between current and voltage. There are also active power and reactive power, whose explanations and correlations with complex power are explained in Table 1 and Figure 1 respectively.

In a power grid, each customer is generally assigned a specific transformer. As the voltage on the supply side is fixed, the voltage on the customer side should be certain and it is called rated voltage. Besides, there are maximum power limits for customers that should not be exceeded. The limit is contracted power (CP) and varies from customer to customer.

Electric parameters are collected at higher frequencies than energy consumption. It not only contains more information about consumption behavior, but also allows us to detect and respond to NTL with lower delay. 

The abnormal consumption that really matters and would cause NTL is the kind that last for a period of time. To be specific, anomalies that take on the forms of impulse signals, sudden changes, or transient data missing, for example, would not make any significant differences to the overall energy consumption (EC) of a customer, and neither would they cause great NTLs. It is a better choice to take them as the consequences of communication failure or temporal metering devices’ malfunctioning and treat the data records from a statistical point of view. 

**Figure 2:**
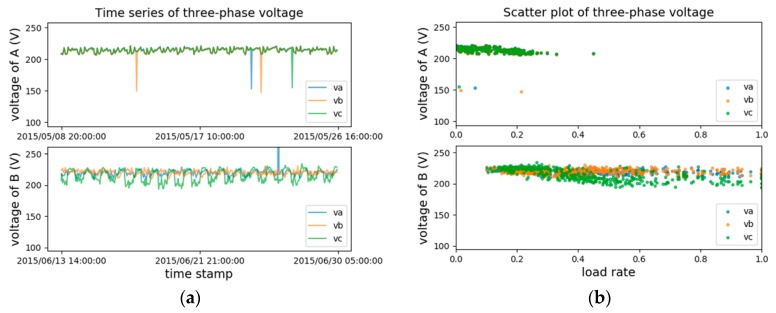
(**a**) Two time series from a normal (upper) and an abnormal (lower) customers respectively. (**b**) The corresponding scatter plots of the same piece of data with load rate being the x-axis. Voltage is collected once an hour and each time stamp is the collecting time.

Figure 2 presents the three-phase voltage records from two customers, normal customer A and abnormal customer B. These two customers are verified by on-field inspections. From the time series in Figure 2a we can see that for the normal customer A (upper), apart from several sudden drops in the single-phase voltage, the voltage values for three phases are highly identical for the rest of the time. However, for abnormal customer B, it is a consistent and almost periodic phenomenon that voltage on phase c differs significantly from the rest of the phases. From the scatter plot of normal customer A in Figure 2b, the several outliers correspond to those impulses in Figure 2a and they behave more like noises in this case. This justifies what was discussed above: focusing on the long term consumption behaviors would help us draw safer conclusions in this NTL detection problem. It is not appropriate to just take each row of data records as a training sample. This is why we proposed our data transformation method. Further experimental comparison between single-collecting-point samples and our statistics-based data representation will be shown in a later section. 

Here, we did not aim to get a precise mathematical representation of the feature distributions. Rather, we designed a data transformation inspired by kernel density estimation (KDE), a non-parametric distribution estimation method. In this work, we did a 2-dimensional KDE transformation of the features obtained in previous section.

The process can be briefly thought of as replacing each discrete point in a scatterplot with a kernel distribution. The idea of this estimation method is that the positions where the points appear have the higher occurrence probabilities. So, for the Gaussian distribution we use as basic kernels, the peaks are where the points are located. The advantage of the distribution profile over a scatter plot is that it gives more intuitive impression of customers’ behavior patterns during a period of time. With the extension of time used for statistical analysis, the estimated distribution becomes more plausible and closer to the scatter plot. 

**Figure 3:**
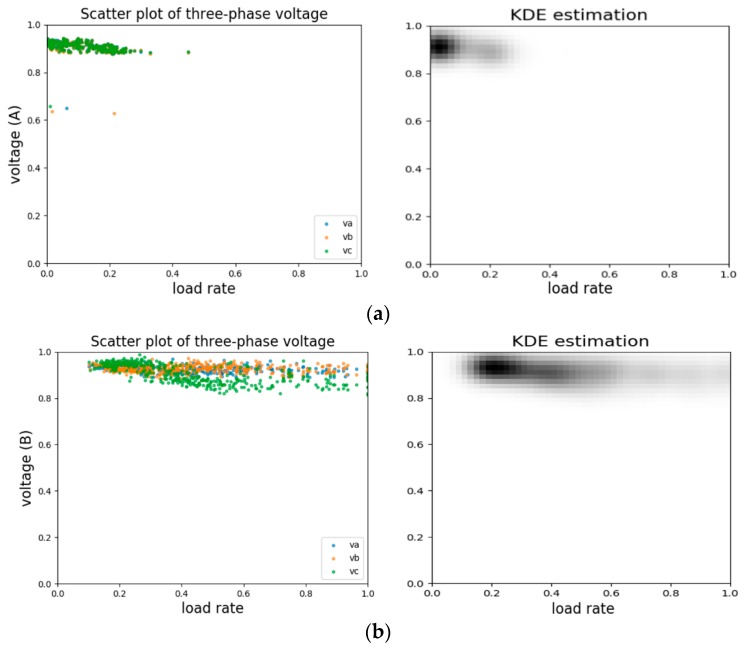
(**a**) Scatter plot (left) and statistical profile using kernel density estimation (KDE) (right) of the normal customer A. (**b**) Scatter plot (left) and statistical profile using KDE (right) of the normal customer B. The voltage value on the y-axis is the raw value divided by customer’s rated voltage.

In this work, we used 2-D KDE to reflect the correlation and variation tendency in the space constructed by two feature indices. As the estimation method is density based, outliers that are rather sparse would have small pixel values thus contributing little to the visual patterns. Figure 3a shows a scatter plot and 2-D KDE profile of the same piece of voltage records data for the afore-mentioned normal customer A. Darker areas in the estimated profile image represents locations where points emerge in high densities and outliers in scatter plot on the left side can hardly be observed in the estimated profile. Technically, the time span needed to generate distribution profile images can vary from sample to sample. If presented with more detailed event annotations as ground truth, we can generate more samples with different time durations as a kind of data augmentation. However, in this situation where specific event logs are not available, it is hard to implement such data augmentations and a fixed time duration of 10 calendar days is adopted throughout this work.

### 2.2. Feature Engineering

The AC voltages and currents are three-phase data. It is fair to treat each of the three phases equivalently because here we only aim to decide whether anomalies/NTL occurred without having to know specifically which phase was going wrong. With regard to the voltage and current, the following features were extracted:

Deviation from rated voltage

(2) $$VD_{i}=\{\begin{array}{cc} \frac{V_{r}-V_{u}^{i}}{V_{r}}, & V_{r}\geq V_{u}^{i}\\ 0, & V_{r}\leq V_{u}^{i} \end{array},$$

(3) $$VD\left(V_{u},V_{r}\right)=max\left(vd_{A},vd_{B},vd_{C}\right),$$

where $V_{r}$ is the rated voltage and $V_{u}^{i}$ is the *i*-th phase voltage. As the voltage should remain steady for a certain customer, the maximum voltage deviation among the three phases from the above formulas can indicate the degree of how abnormal a customer is.

Unbalance degree of voltage and current on three phases

(4) $$AVG=mean\left(S_{A},S_{B},S_{C}\right),$$

(5) $$UD=\frac{mean\left(abs(S_{A}-AVG\right),abs\left(S_{B}-AVG\right),abs\left(S_{C}-AVG\right))}{AVG},$$

where *S* can either be voltage or current.

Unlike the voltage, there exists no such standard value for the current. However, the discrepancy of voltage or current values in three different phases should not be too large if the system is running as expected. After all, any significant value decrease in any phases would directly result in the drop of EC. Formula (5) well represents the difference of voltage or current in three phases.

*Active Power.* Although there are many different types of customers and their real-time active power collected once an hour may vary greatly, we can use the contracted power to normalize it to make the power feature comparable among customers.

*Power factor.* This feature is customer-invariant and has the value range from 0 to 1. 

*Load rate.* It indicates the percentage that the power accounts for of the maximum power volume. The formula is more like an approximation of the apparent powers normalized by the contracted power. In most of the 2-dimensional distribution profile image channels, we used the load rate as the reference axis *x*.

(6) $$load\;rate=(\left|I_{a}\right|+\left|I_{b}\right|+\left|I_{c}\right|)*V_{r}/CP,$$

### 2.3. Generation of Super Images

**Figure 4:**
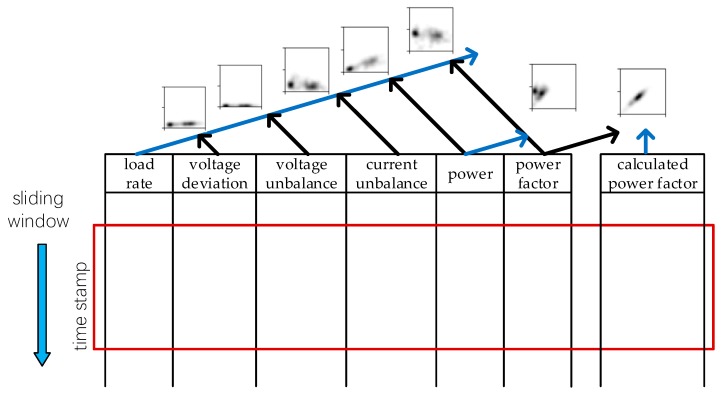
The generation of the seven-channel super image. Each image channel is generated with a combination of the data columns shown in the figure. Blue lines indicate the columns of features used as x-axis data while black lines indicate the column used as y-axis data. The rectangular box in red is the sliding window that covers a certain time range of data. Here we have another feature named calculated power factor. This is the power factor we calculated with the power and load rate values.

We used a Gaussian kernel with fixed standard deviation σ and formed images as shown in Figure 3. Notably, with all the features mentioned above, we can use combinations of them to get short time distribution profiles of these feature pairs. Seven grey-scale maps are generated altogether as described in Figure 4. With each of these maps constituting one channel, a super image with seven channels is obtained and this is the input for the detecting neural network. Multiple samples can be generated from a customer by having a sliding window of fixed size move along the time axis of a customer’s data records. The comparison between samples from statistical profile and per-row record will be shown in later section in terms of separability and final detection performance.

Before feeding the super image to the network, we have to search the bounding boxes of patterns in each image channel. Position information obtained in this stage serves as auxiliary input for the network to highlight the position of the pattern objectives. We achieved this goal by setting a pixel threshold, and pixel values higher than the threshold are included in the bounding box. The choice of threshold value is listed in Appendix B. 

### 2.4. Framework

The on-field inspection requires great deal of human efforts, so the number of verified customers is quite limited. Semi-supervised learning distinguishes itself by utilizing both the labeled and unlabeled data. There are different fashions of implementing the goal: self-training [12], co-training [13], transductive SVM [14], graph based methods [15], and generative models [16]. With the development of deep neural networks, novel models such as generative adversarial nets (GAN) [17] and tons of its variant versions emerged. The idea of GAN is brilliant and GANs showed their superiority in various tasks. However, GANs usually require prior knowledge about the label distribution of the unlabeled samples. There is also other previous research that made significant progress in this branch of semi-supervised learning, like Virtual Adversarial Training (VAT) [18,19] and ladder network [20]. These methods either explicitly or implicitly depend on prior knowledge about the distribution of labels. As we have no idea about the label distribution in unlabeled data, we have to utilize semi-supervised learning in a way that is independent of such prior knowledge. In this work, we used consistency regularization based techniques [3,21,22,23] to train our model. Throughout the paper, the ensemble strategy we adopt is from the mean teacher model [3]. 

**Figure 5:**
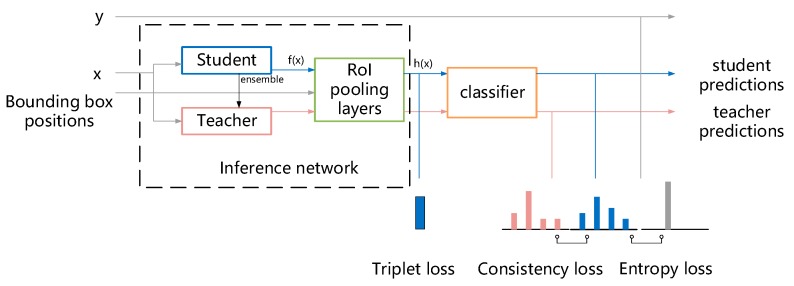
Overview of the entire model structure. The student model and teacher model have the same network architecture while the parameter values like weights and biases are different. Parameter values of student model are updated at the end of each training iteration and those of the teacher model are obtained by ensembling the student model. What triplet loss does is minimize the feature distances between input with the same labels while maximizing the feature distances of input with different labels. For the unlabeled samples, labels needed for computing triplet loss are from the teacher model’s predictions.

In most cases, overfitting results from the scarcity of labeled training samples. What semi-supervised learning does is utilize unlabeled samples to prevent overfitting. If there exists an oracle that can give the labels of unlabeled samples, we can introduce a regularization term to the optimization objective to constrain the model and make the model give identical predictions with the oracle. In the mean teacher model, such an oracle is called teacher model in this case. It is obtained by ensembling the student model. The prediction it yields is called a pseudo label. Although the pseudo labels are not perfectly correct, as training goes on, the student model becomes better and better and the pseudo labels are more and more reliable as a result. It is much like a student that would learn well with the right instructions from the teacher. In turn, a good student would make the teacher improve. Gradually, both the student and teacher would become excellent in the end. Formula (7) gives the mathematical forms of how ensembling is implemented. And Figure 5 is the overall framework of the model. 

### 2.5. Inference Network

**Figure 6:**
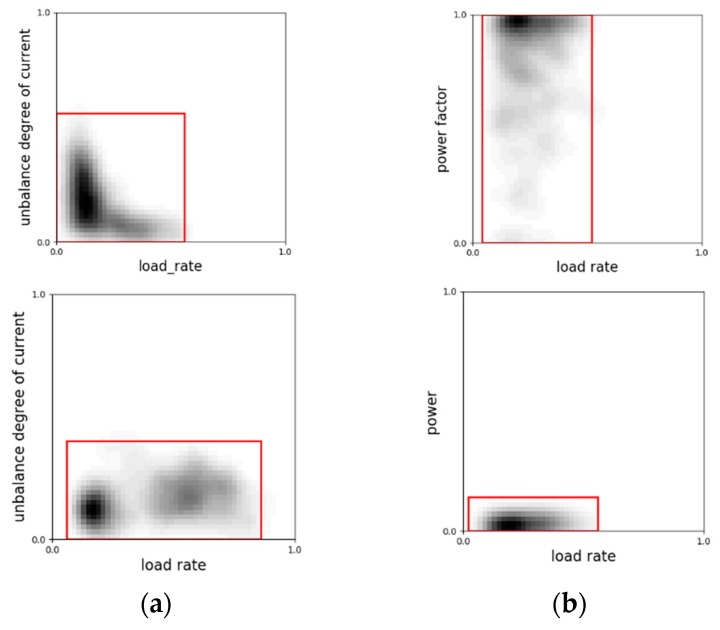
(**a**) The distributions of the 2-D points (load rate, current unbalance degree) from a normal (upper) and an abnormal (lower) customer. (**b**) The distributions of 2-D points (load rate, power factor) and (load rate, power) from the same abnormal user. The ranges are marked with red bounding boxes. Normally, the unbalance degree decreases as the load rate grows as shown in the left in (**a**). As the current on three phases grows, small disturbance would not have much effect on it. So the unbalance degree would turn low and steady. The right figure contradicts this fact. In (**b**), the power factor is 1 for the most of the time, but the power data is nearly zero. This is a typical sign of customers conducting theft.

**Figure 7:**
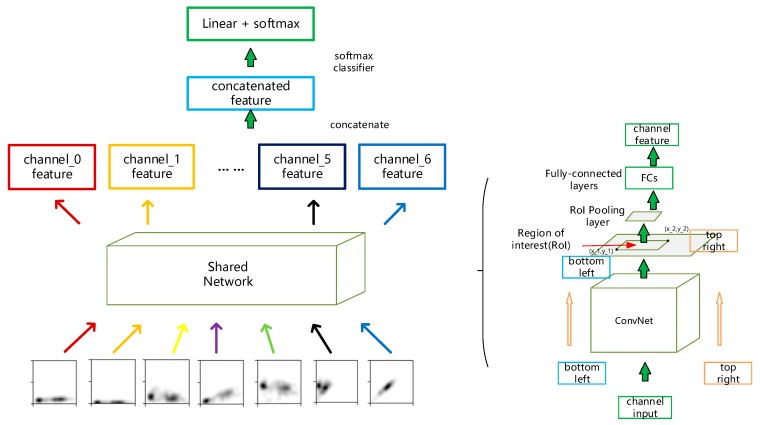
The structure of the inference network.

We get the distribution of features to expose their visual patterns for the model to capture. For this job, convolutional based neural network has already proved its superiority and has achieved remarkable results in various image recognition and computer vision tasks [24]. Also, there exists previous work that encoded time series as images to achieve improvement encoding/decoding problems [25]. For visual object detection problems, there are a series of famous works about two-stage object detection [26,27,28]. The regions where objects lie in an image are proposed by bounding boxes for further classification. Our task shares the same purpose of highlighting the desired consumption pattern, so these previous works serve as great examples of how to classify objects that occupy only part of the entire image. Figure 6 gives a detailed example of how we can decide a sample is abnormal or not. The structure of our inference network is illustrated in Figure 7.

Figure 7 is the overall structure of our inference network. Although each statistical profile is treated as a channel of a super image, the information each of these channels carries, such as pattern shapes and the regions patterns lie in, are totally different and should be processed separately. Every channel in a super image goes through a shared network whose internal structure is shown in Figure 6 (right). The internal structure of the shared network is much like that in [27] except that we search the region of interest here by setting a pixel value threshold to get a rectangular area where the pattern lies. So there is only one bounding box for an input channel. After obtaining every channel feature, we concatenate them together to get a final feature representation of an input super image. The convolutional based network denoted as ‘ConvNet’ used the same set of parameters to extract graphic information of all the image channels and its detailed architecture is listed in Appendix A Table A1.

### 2.6. Ensembling Method and Consistency Loss

Formally, let θ denote the weight parameters in the neural network and $\theta_{t}$ denote the parameters at training step t. The ensembling strategy is as follow:

(7) $$\theta_{t}^{\prime }=\alpha \theta_{t-1}^{\prime }+\left(1-\alpha \right)\theta_{t},$$

Then, consistency cost is introduced to minimize the prediction difference between the teacher and student models:

(8) $$J\left(\theta \right)=E_{x,\eta^{\prime },\eta }\left[\left\|f\left(x,\theta^{\prime },\eta^{\prime }\right)-f\left(x,\theta ,\eta \right)\right\|\right],$$

where, η is the noise applied to input image.

Apart from the consistency cost, we introduced another loss item, triplet loss [29]. This is originally proposed for face recognition tasks. However, the goal of face verification is more demanding than general classification problems and what triplet loss does is to constrain points of same classes to have consistent embeddings while pushing points of different classes far away in the embedding space. Let $x_{i}$*,* $x_{j}\;$ be two input samples with index *i* and *j*. Let *y* and *h* denote the label and extracted features respectively. Equation (8) is an explanatory formula of how triplet loss works:

(9) $$l_{G}\left(i,j\right)=\{\begin{array}{cc} {\left\|h\left(x_{i}\right)-h\left(x_{j}\right)\right\|}^{2} & if\;y_{i}=y_{j}\\ max\;(0,margin-{\left\|h\left(x_{i}\right)-h\left(x_{j}\right)\right\|}^{2}) & if\;y_{i}\neq y_{j} \end{array}$$

Additionally, the labels in the formula are not all ground truthed in the labeled data set and the triplet loss is applied for all the input in a training batch. For those input without labels, we use the predictions given by the teacher model as pseudo labels.

### 2.7. Training Algorithm

With the framework and the loss items introduced, the pseudo code of our training algorithm is presented in Algorithm 1. Notably, the coordinates of bottom left and top right vertices of bounding boxes are sent to the network as auxiliary input. The Region of Interest (RoI) pooling operation is executed on the feature maps and the coordinates of bounding boxes are linearly projected on the feature map. As for our strategy of drawing mini-batches, we separately draw samples from labeled data set and unlabeled data set by random while maintaining the ratio of 1:3 between labeled and unlabeled samples.
**Algorithm 1.** Training algorithm.**Require**: $x_{i}$ = input images**Require**: *L* = set of training input indices with known labels**Require**: $y_{i}$ = labels for labeled inputs, *i* ∈ *L***Require:**$bb_{i}$ = bounding box of sample *i***Require:***W_u* = unsupervised loss weight**Require:**$f_{\theta }\left(x\right)$ = neural network with trainable parameters θ as student model**Require:**$f_{\theta^{\prime }}\left(x\right)$ = neural network as teacher model whose parameter $\theta^{\prime }$ with initial value θ**Require:**α = moving average momentum for parameters**Require:**η = random Gaussian noise added to the input**for***t* in [1, num_iterations] **do**draw a mini-batch B from labeled and unlabeled samples randomly$f_{i}\leftarrow f_{\theta }\left(x_{i\in B},bb_{i\in B},\eta \right)$ evaluate network outputs$f_{i}{}^{\prime }\leftarrow f_{\theta^{\prime }}\left(x_{i\in B},bb_{i\in B},\eta^{\prime }\right)$ evaluate network outputs Find triplets *T* of components <*i*, *j*, *k*> in B where $y_{i}=y_{j}$ and $y_{i}\neq y_{j};\;y_{i}$ is the pseudo label given by $f_{i}^{\prime }$ if i∉(B∩L) $loss\leftarrow -\frac{1}{\left|B\cap L\right|}\underset{i\in \left(B\cap L\right)}{\sum }y_{i}logf_{i}$*+*$W\_u\left(\frac{1}{\left|\left\{i|i\notin \left(B\cap L\right)\right\}\right|}\underset{i\notin \left(B\cap L\right)}{\sum }{\left\|f_{i}-f_{i}^{\prime }\right\|}^{2}\right)$*+*$W\_u\left(\frac{1}{\left|T\right|}\underset{i,j,k\in B}{\sum }l_{G}\left(i,j\right)+l_{G}\left(i,k\right)\right)$update θ using ADAM optimizerupdate $\theta^{\prime }$ by $\theta_{t}^{\prime }=\alpha \theta_{t-1}^{\prime }+\left(1-\alpha \right)\theta_{t}$**end for**return θ

## 3. Results

### 3.1. Experiment Settings and Metrics

The electric parameters used in our experiment are from real-world SM records. We use the data from a power company in China to train and validate our model. The detailed information of our dataset is shown in Table 2. There are 193 verified and 2929 unverified customers in total, among which 54 of them are labeled as abnormal customers by on-field inspection, whatever the causes are, and 139 of them are labeled as normal ones. As is introduced in Section 2, data records of a customer can be transformed into numbers of samples as the sliding window moves along the time axis. Here we adopt the sliding window of a 10-day time range regardless of the features’ sampling frequency, and the overlap between windows is five days, that is to say, each image channel of a sample consists of 240 points if the corresponding feature is collected hourly. As a result, it would make 8797 labeled training samples and more than 130,000 unlabeled samples in total. However, in order to significantly cut down time for training, we randomly selected 50,000 samples from part of the unlabeled users. In order to justify the generalization ability of our method, we split the verified customers randomly while maintaining the ratio between the number of customers in training and validation sets, which is approximately 1:3. This process is repeated for three times. More experiments settings are listed in Appendix B.

For the detection problem where the negative samples are far more than positive ones, overall precision is not a suitable criterion to assess the performance of our algorithm. However, the precision and recall of the positive samples alone would give a direct impression of the detection results.

As is in most binary classification problems where the output is a two-dimensional vector that gives the corresponding probability of each class the input belongs to, we take the hypothesis with higher probability as the prediction. In other words, the decision threshold is 0.5. When the threshold varies, the accuracy and the sensitivity, true positive rate (TPR) and false positive rate (FPR) of the classifier also change. Generally speaking, classifiers with high TPR and low FPR are excellent for classification problems.

### 3.2. Separability

**Figure 8:**
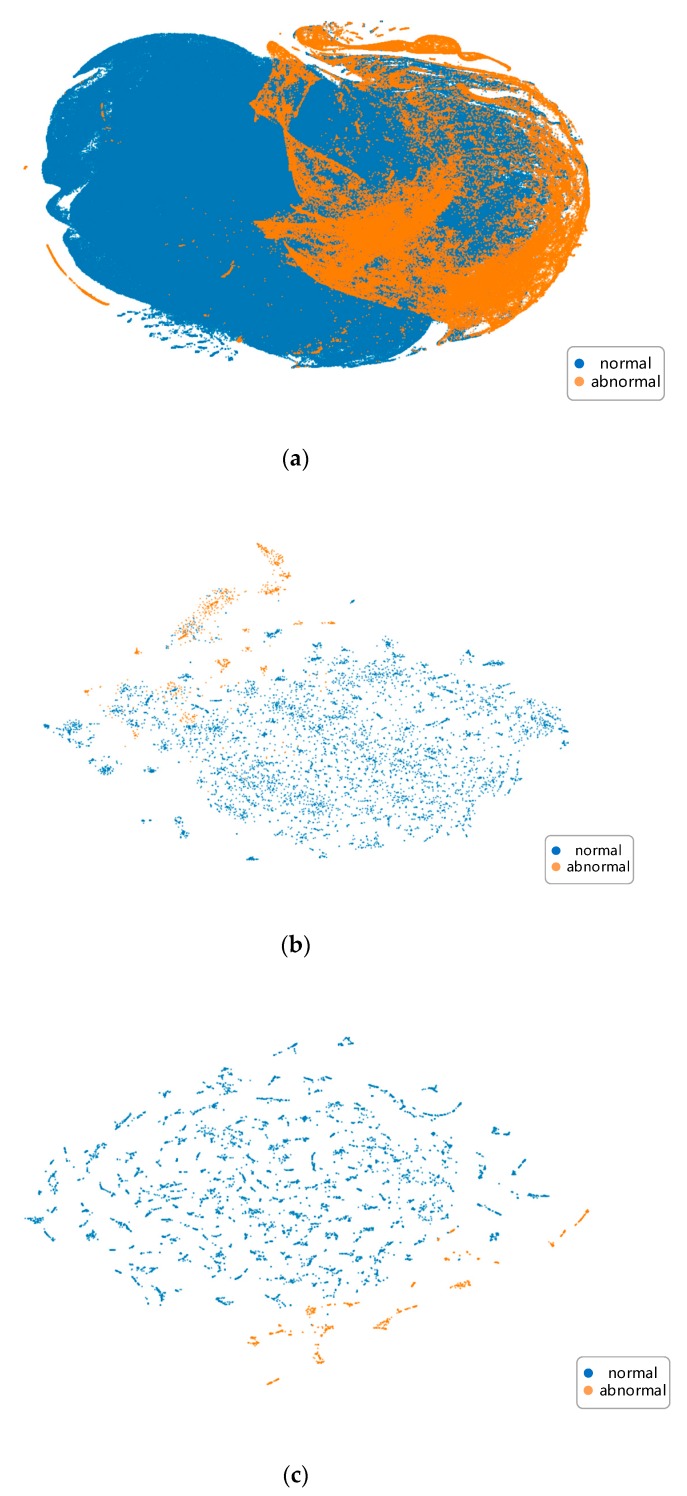
t-SNE visualization comparison of different ways of generating data samples. Points in orange and blue indicate abnormal and normal types respectively. Notably, the number of points in (**a**) is much more that those in (**b**,**c**). This is because each sample in (**b**) contains a certain time range of raw features in (**a**).

To justify our method of transforming the data, we visualized linear separability of the training data using a visualization technique called t-distributed stochastic neighbor embedding (t-SNE) [30]. It non-linearly projects features from high-dimensional space to lower-dimensional space while trying to maintain their original relative positions. As shown in Figure 8a is the 2-D projection of the 6-D feature vector (load rate, voltage deviation, voltage unbalance degree, current unbalance degree, power factor, and power) and each point represents the features at one sampling time stamp in the validation set. In Figure 8b, we use the same features but take a longer time range of 10 days to form each point in the figure. Points in Figure 8c are our proposed seven-channel ‘super images’ generated from a 10-day time range of features.

Obviously, our transformation method makes the classification problem more feasible as the result in Figure 8a shows that it is nearly impossible to separate samples of different labels. This is an indication that viewing the data in the longer term is a better solution to address the detection problem.

**Figure 9:**
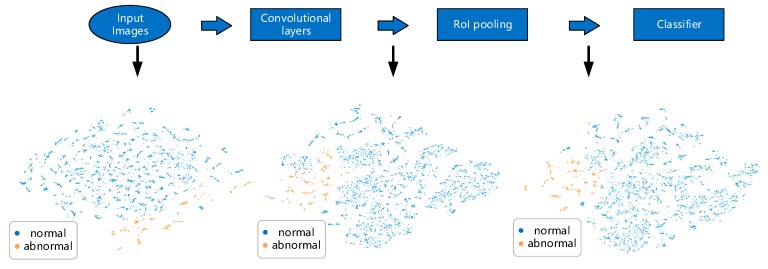
Linear separability of samples in validation set changes as samples go through the inference network.

From Figure 9 we can see that as samples go through the inference network, points of the sample labels gradually gather together, especially for the abnormal samples which are plotted in orange. This is further evidence that our data transformation, together with such network structure, works well for our classification goal. 

### 3.3. Detection Results

Verified customers were randomly split into a training set and validation set for three separate times and the training and validation process is carried out based on the data. Detection accuracy was not suitable to assess our algorithm performance because in this situation where negative samples are in absolute majority, the overall accuracy would still seem rather high even if our algorithm fails to find out any positive samples. As a result, receiver operation characteristic (ROC) and area under curve (AUC) [31] score as well as precision–recall (PR) curves are used as assessment criteria.

Similar to any other anomaly detection scenarios, our data set is quite imbalanced where most of the data is negative/normal in both training and validation set. So apart from overall performance, we are particularly interested in our algorithm’s performance on positive/abnormal samples. 

**Figure 10:**
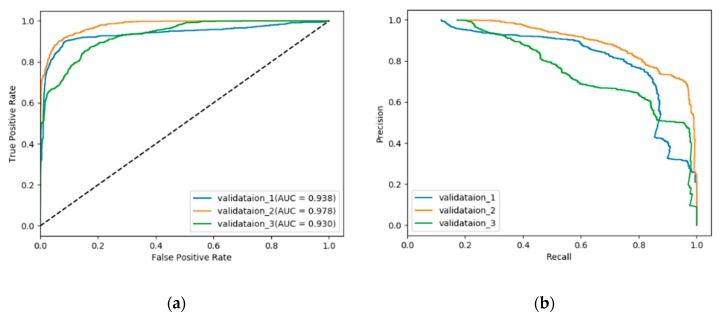
(**a**) Receiver operation characteristic (ROC) curves (**b**) precision–recall curves in three experiment rounds on different training/validation sets.

Figure 10 and Table 3 give the results from a single run of each train/validation splits. The mean and standard deviations report of each validation set is available in Appendix C.

For our NTL detection problem, the characteristics of data records from so many customers are very different because the variety of types of customers. This fact can be directly reflected in the detection results shown in Figure 10. For different train/validation splits, differences in terms of ROC curves, AUC score or precision–recall curves are all non-negligible. This is totally understandable and we suppose it is due to our random selection strategy: sometimes the customers chosen for training can well cover most of the abnormal causes of NTL while sometimes the customers for training have only limited pattern of manifestation. However, all the detection results are pretty good from multiple perspectives, which demonstrates that this method has its specialties.

Our forward inference network consisted of nine convolutional layers, which is denoted Conv9 in Table 4. We justify that utilizing the unlabeled samples is essential by comparing the results of models trained with or without the semi-supervised learning strategy in Table 4. Even though we used some sampling strategy to make sure that in a training batch the ratio of positive and negative samples was kept constant throughout the experiments, results show that sole labeled training data is not enough for a model to perform well on a larger and maybe more complicated validation sample set. To guarantee that our inference network is not the cause of the poor performance of supervised model, we used a more traditional and reliable architecture, resnet-21. It turns out it still cannot address the problem that a supervised model basically classifies every sample as negative. 

**Figure 11:**
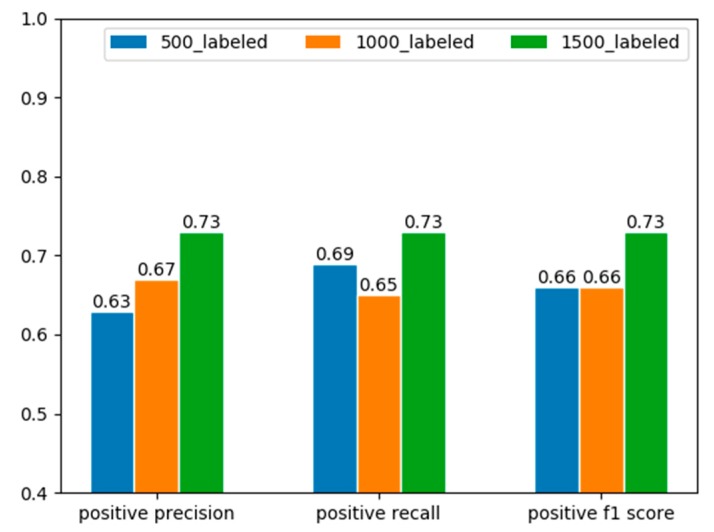
The precision, recall and f1 score of positive samples on the same validation set as the number of labeled samples changes. The results are obtained by randomly selecting part of the total labeled training samples and use the same model and semi-supervised training method.

Semi-supervised learning methods help find a more reasonable decision boundary with the presence of unlabeled data thus preventing the overfitting problem caused by the limited labeled data. Figure 11 gives the results when we change the size of labeled data in training set while keeping other settings unchanged. Generally speaking, the anomaly detection performance gradually increases as the size of labeled samples grows from 500 to 1500. There is a slight drop in recall rate when the size of labeled data changes from 500 to 1000, but the precision increased a bit. 

In order to prove that our network architecture is different and more suitable for the detection problem and especially for our designated data format, we did a comparison with the original mean teacher model by gradually adding our adaptations. Also, in order to justify the proposed the network structure, we trained SVMs (with balanced class weights) as the baseline classifier and chose the result with highest f1 score by ranging the penalty parameters from 0.01 to 1000 (increasing by orders of magnitude). We used the original mean teacher model and gradually added improvements on top of it. The results tested on different models are shown in Table 5.

For the general NTL detection goal, it is equivalently important have a high value on both precision and recall. Extreme result on either single index is obviously not desired. Generally, SVM does a great job in terms of recalling the NTL samples but the detection accuracy is apparently much lower. As for following the models, simpler models tend to categorize samples into the class that is in the majority. An intuitive explanation of such improvement is that triplet loss tries to minimize the pattern discrepancies among users in a same category while the role RoI pooling plays is that it highlights where the points cloud is in an image channel and naturally makes the pattern features more explicit. We can infer that our network architecture does improve the performance of detection.

### 3.4. Discussion

The major challenges for most of the NTL detection problems are that the anomalies can take on various forms of manifestations that are hard to capture. Worse still, the number of cases we can study from is often limited as a result of the labor-consuming on-field inspection. So it not a traditional anomaly detection problem and it is essential to make use of all the types of data available to get complete and prominent representations of the input. 

Most of the previous works mainly focus on the historical energy consumption data because it has the most direct relevance to NTL detection problem. However, the types of NTL that can be detected are bound to be limited by the limited choice of measurements. [4,5,6,7,8] used sole energy consumption (EC) data. Apart from it, [32] utilized auxiliary databases and [33] made use of credit worthiness ratings. In [34], researchers tried to gather different features from various types of SM data such as the quality of measurements, electrical magnitudes, GIS data, and technological characteristics of the SM besides EC. As for our proposed method, we utilize as many types of electrical parameters we can access. There are two main advantages of our data transformation method: it focuses on the customers’ long term behaviors rather than single data points; the multi-channel image input makes it possible for the deep neural networks to automatically extract the underlying correlations among these features. Besides, our data transformation approach can tolerate slight data distortion or absence. We did not make use of specific auxiliary data except the most common electrical parameters. Such transformation provides a new aspect of viewing the electrical parameters.

Furthermore, our proposed approach deals with the situation where there are only a small number of labeled samples. Table 6 shows the comparison between some of the state-of-the-art approaches and our proposed approach. All these approaches except ours deal with the problem in the supervised fashion which took advantage of the abundance of customer class information. Also, Figure 11 shows that although the detection performance may suffer with the decrease of the number of labeled samples, acceptable results can still be obtained. To our limited knowledge, our work is the first to solve the NTL detection problem with a semi-supervised method.

Another advantage of our method is that the time range we need to generate a sample is rather short compared with other approaches listed in Table 6. The approach in [11] can make judgments with no delay but actually it is achieved by comparing the current consumption with the average of consumption in the past, so it is not technically real-time. Apart from [11], the detection delay of our methods is apparently shorter. This allows us to make quicker responses to the emergent NTL attacks. For a trained model, we can use a certain range of data records to form our data sample to sufficiently cut down the detection delay. 

As for data privacy, the transformation we did hides the original information that SM collected. So there is nearly no original information exposed. 

There are still some weaknesses or works we have not done sufficiently for now. Even though we aim to cover as many types of anomalies as possible in the detecting stage, we have not come up with a solution to explicitly indicate the exact causes of NTLs. Our detection results strongly depend on the coverage of different forms of NTL attacks used in the labeled training set.

## 4. Conclusions

This paper presented a thorough methodology for detecting non-technical losses. The transformation from the data records collected by smart meters into the super images allows us to view the consumption behaviors of a customer from a statistical perspective in the longer term. The new data format also provides a different way of extracting features and integrated analysis of more types of features would have greater potential of detecting a wider range of anomalies. Followed by the prior knowledge free semi-supervised learning strategy, our method demonstrated its superiority to the supervised learning in the situation where labeled data is in the minority of the entire data set.

Our method is trained and validated on the real data from a power grid of China. With reference to some ideas in two stage object detection models, we designed our network architecture to effectively capture features for classification. Ablation studies in the experimental section demonstrate that our method in each stage does work and the comparison with the state-of-the art methodologies proves that our result is rather competitive and our method has its advantages.

## Figures and Tables

**Table 1:** Parameters.

Items	Notes
$V_{a},V_{b},V_{c}$	Voltage on three phases
$I_{a},I_{b},I_{c}$	Current on three phases
Active power	Power that does work
Reactive power	Power that does not do any work
Power factor	Cosine of the phase of voltage relative to current

**Table 2:** Description of data set.

Customer Label	Number of Customers	Number of Data Records	Number of Samples
Normal	139	1,145,832	7685
NTL detected	54	137,112	1112
Unlabeled	2929	16,390,248	132,481

**Table 3:** Precision and recall of positive samples.

Experiment #	Precision	Recall	F1-Score	Overall AUC Score	Positive/Total
Validation_1	0.84	0.74	0.79	0.9379	785/6691
Validation_2	**0.97**	**0.88**	**0.92**	**0.9775**	744/6476
Validation_3	0.84	0.80	0.82	0.9304	835/6658

**Table 4:** Comparison of semi-supervised and supervised learning methods.

Model	Label	Precision	Recall	F1-Score
Conv9 + semi-supervised	Normal	**0.97 ± 0.0047**	0.98 ± 0.0047	**0.97 ± 0.0047**
	NTL	0.88 ± 0.061	**0.81 ± 0.057**	**0.84 ± 0.056**
Conv9 + supervised	Normal	0.88 ± 0.00	**1.00** ± 0.00	0.94 ± 0.00
	NTL	0.00 ± 0.00	0.00 ± 0.00	0.00 ± 0.00
Resnet-21 + supervised	Normal	0.89 ± 0.0047	**1.00** ± 0.00	0.94 ± 0.00
	NTL	**1.00** ± 0.00	0.02 ± 0.0082	0.043 ± 0.017

**Table 5:** Results by different model settings.

Model	Label	Precision	Recall	F1-Score
SVM	Normal	0.99	0.94	0.96
	NTL	**0.67**	**0.96**	**0.79**
Mean teacher	Normal	0.91 ± 0.00	1.00 ± 0.00	0.95 ± 0.00
	NTL	0.88 ± 0.00	0.23 ± 0.025	0.36 ± 0.029
Mean teacher + Triplet loss	Normal	0.94 ± 0.0047	0.98 ± 0.0047	0.96 ± 0.0047
	NTL	0.72 ± 0.022	0.48 ± 0.022	0.5 ± 0.022
Ours	Normal	**0.97 ± 0.0047**	**0.98 ± 0.0047**	**0.97 ± 0.0047**
	NTL	**0.88 ± 0.061**	**0.81 ± 0.057**	**0.84 ± 0.056**

**Table 6:** Comparison with the state-of-the-art methods.

Criteria	[32]	[9]	[33]	[11]	[34]	Ours
Data privacy	low	high	high	medium	medium	high
Data types	Monthly EC	Monthly EC and auxiliary databases	Monthly EC and auxiliary databases	Monthly EC and auxiliary databases	SM data and auxiliary databases	Hourly SM data
Detection delay	12 months	12 months	12 months	-	90 days	10 days
AUC score	0.56	0.63	0.74	0.84	0.91	0.94

## Appendix A. Architecture of the Convolutional Based Layers

**Table A1:** Architecture of the convolutional based layers.

Input 50 × 50 × 1 (One Channel of a Super Image)
Gaussian noise σ = 0.15
3 × 3 conv. 32 lReLU (α=0.1) same padding followed by Batch Normalization (BN)
3 × 3 conv. 32 lReLU (α=0.1) same padding followed by BN
2 × 2 max-pool, dropout 0.5
3 × 3 conv. 64 lReLU (α=0.1) same padding followed by BN
3 × 3 conv. 64 lReLU (α=0.1) same padding followed by BN
2 × 2 max-pool, dropout 0.5
3 × 3 conv. 128 lReLU (α=0.1) same padding followed by BN
3 × 3 conv. 128 lReLU (α=0.1) same padding followed by BN
2 × 2 max-pool, dropout 0.5
3 × 3 conv. 256 lReLU (α=0.1) same padding followed by BN
1 × 1 conv. 128 lReLU (α=0.1) same padding followed by BN
1 × 1 conv. 64 lReLU (α=0.1) same padding followed by BN

## Appendix B. Experiment Settings

**Input images.** Input images are generated according to the approaches introduced in Section 2.3. And we set the image resolution to 50 × 50. No augmentation methods are applied throughout the experiments. And the pixel threshold to search the bounding boxes is 5.
**Framework and Devices.** Models mentioned in this paper are implemented using Tensorflow 1.0.0. He initialization [35] is used on all layers. Three 1080ti GPUs are utilized to achieve parallel data processing, with each GPU device taking a batch of input data of size 36.
**Optimizer.** Adam optimizer with the parameter betTABLE as 0.9 and beta2 as 0.999. In each iteration batch input of size 108 (36 × 3) are fed into the network.
**Training schedule.** Fixed learning rate of 0.003 are adopted throughout the experiments. Unsupervised loss weight *W_u* introduced in Algorithm 1 is set to a fixed value of 0.18. And the margin introduced in Equation (9) is 1.0.

## Appendix C

**Table A2:** The Mean and Standard Deviations Report of Precision and Recall of Positive Samples (Three Runs).

Experiment #	Precision	Recall	F1-Score	Positive/Total
Validation_1	83.86% ± 0.48%	73.25% ± 0.84%	78.19 ± 0.58%	785/6691
Validation_2	**96.93 ± 0.36%**	**87.68% ± 0.28%**	**92.07% ± 0.20%**	744/6476
Validation_3	83.98% ± 0.21%	79.56% ± 0.31%	81.71% ± 0.19%	835/6658
